# mRNA translation is a therapeutic vulnerability necessary for bladder epithelial transformation

**DOI:** 10.1172/jci.insight.144920

**Published:** 2021-06-08

**Authors:** Sujata Jana, Rucha Deo, Rowan P. Hough, Yuzhen Liu, Jessie L. Horn, Jonathan L. Wright, Hung-Ming Lam, Kevin R. Webster, Gary G. Chiang, Nahum Sonenberg, Andrew C. Hsieh

**Affiliations:** 1 Division of Human Biology, Fred Hutchinson Cancer Research Center, Seattle, Washington, USA.; 2 Department of Urology, University of Washington, Seattle, Washington, USA.; 3 Cancer Biology, eFFECTOR Therapeutics, San Diego, California, USA.; 4 Department of Biochemistry, McGill University, Montreal, Quebec, Canada.; 5 University of Washington Departments of Medicine and Genome Sciences, Seattle, Washington, USA.

**Keywords:** Oncology, Mouse models, Translation, Urology

## Abstract

Using genetically engineered mouse models, this work demonstrates that protein synthesis is essential for efficient urothelial cancer formation and growth but dispensable for bladder homeostasis. Through a candidate gene analysis for translation regulators implicated in this dependency, we discovered that phosphorylation of the translation initiation factor eIF4E at serine 209 is increased in both murine and human bladder cancer, and this phosphorylation corresponds with an increase in de novo protein synthesis. Employing an eIF4E serine 209 to alanine knock-in mutant mouse model, we show that this single posttranslational modification is critical for bladder cancer initiation and progression, despite having no impact on normal bladder tissue maintenance. Using murine and human models of advanced bladder cancer, we demonstrate that only tumors with high levels of eIF4E phosphorylation are therapeutically vulnerable to eFT508, the first clinical-grade inhibitor of MNK1 and MNK2, the upstream kinases of eIF4E. Our results show that phospho-eIF4E plays an important role in bladder cancer pathogenesis, and targeting its upstream kinases could be an effective therapeutic option for bladder cancer patients with high levels of eIF4E phosphorylation.

## Graphical abstract

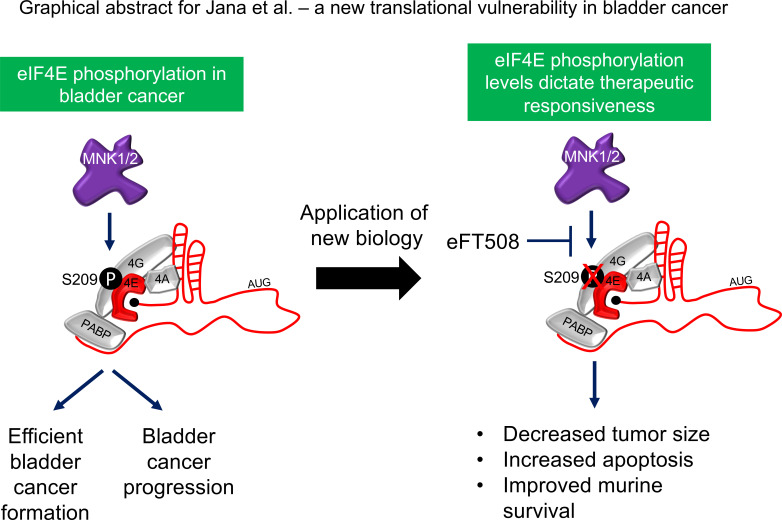

## Introduction

Bladder cancer is predicted to have afflicted 81,400 individuals within the United States in 2020. Furthermore, it is estimated that 17,980 men and women will have died from the disease in the same year (1). While new therapeutics including checkpoint blockade agents (2), FGFR inhibitors (3), and antibody drug conjugates (4) have recently been approved for patients with highly aggressive urothelial carcinoma, metastatic disease remains fatal for the majority of patients, despite a growing wealth of genomic data, which has expanded our understanding of lethal disease (5–8). Therefore, new biology uncovering previously unrecognized therapeutic vulnerabilities is needed. Protein synthesis is a fundamental molecular process that is critical for cancer initiation and progression (9–13). However, it is unknown whether intact protein synthesis is necessary for normal bladder development and maintenance or the natural history of bladder cancer initiation and progression. These are critical questions because precisely targeting the translation apparatus is becoming increasingly possible through the development of targeted therapeutics with physiologic efficacy in patients (14–16). As such, understanding the protein synthesis requirements of the normal bladder and how it is perturbed in urothelial cancers represents a potential new treatment paradigm.

Here, we demonstrate that mRNA translation is a requisite for efficient bladder urothelial cell transformation but is dispensable for normal bladder epithelial development and maintenance in vivo. Through a candidate gene analysis of key translation regulators, we determine that phosphorylation of the oncogene eukaryotic translation initiation factor 4E (eIF4E) is significantly upregulated in the context of bladder tumor formation. Utilizing an eIF4E S209–knock-in mouse model, we show that this posttranslational modification is not only associated with transformation, but is also necessary for efficient tumorigenesis. The sole kinases responsible for eIF4E S209 phosphorylation are MAPK interacting serine/threonine kinase 1 (MNK1) and MNK2 — and eFT508, a selective inhibitor of MNK1 and MNK2, was recently developed and is currently being evaluated in trials (14, 17, 18). Using this compound along with murine- and human-derived organoid models and patient-derived xenografts (PDX), we demonstrate that eIF4E phosphorylation is necessary for bladder cancer progression and is a requirement for the therapeutic response to eFT508, which represents a potentially new therapeutic vulnerability in bladder cancer that can extend survival.

## Results

### Optimal protein synthesis is required for efficient urothelial transformation.

**Figure 1:**
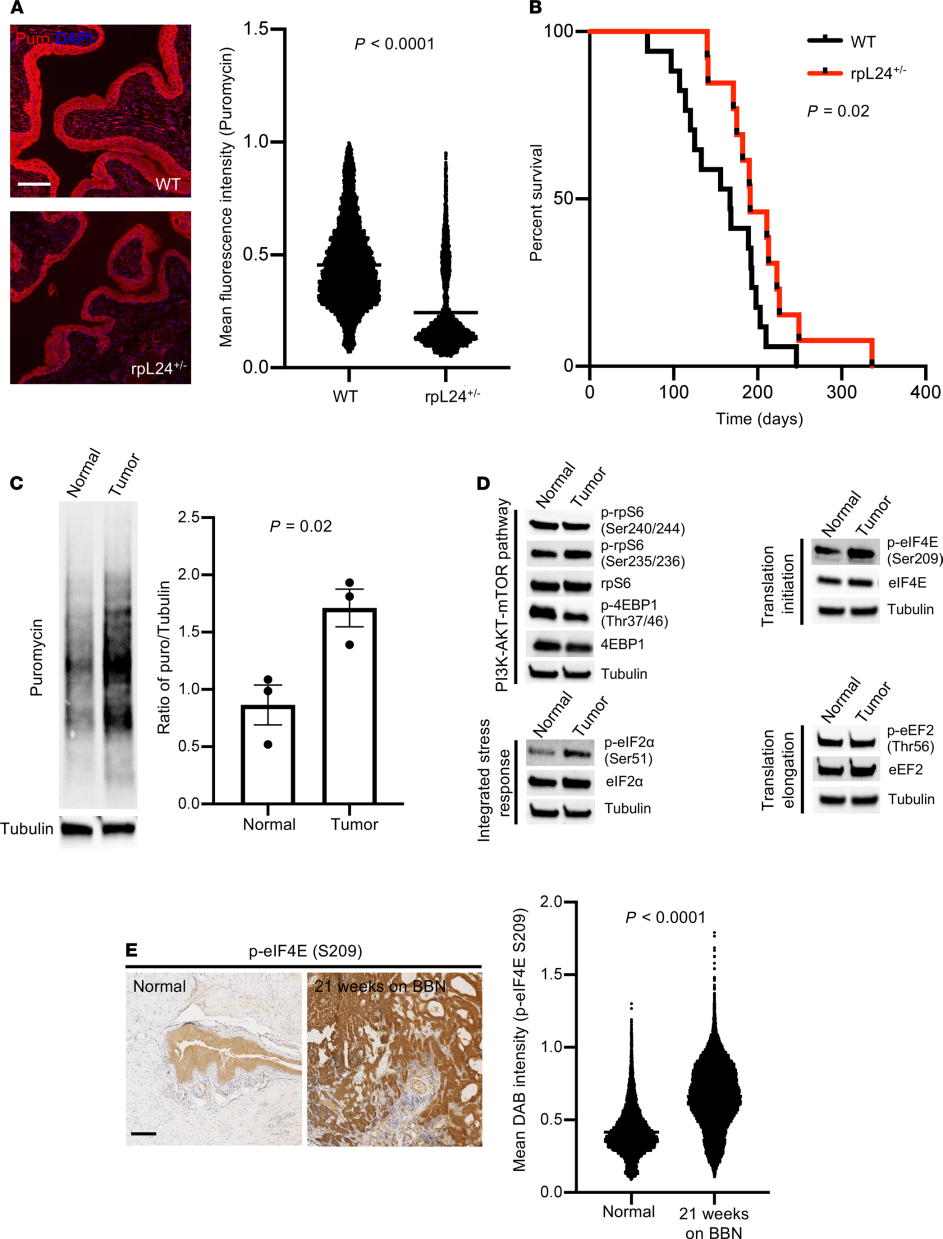
Optimal protein synthesis is necessary for efficient urothelial cell transformation in vivo, and eIF4E phosphorylation selectively increases in the context of bladder cancer formation. (**A**) Puromycin incorporation in WT and rpL24^+/–^ urothelium. Representative IF images show less protein synthesis in rpL24^+/–^ mice compared with WT counterparts. Quantification of > 5000 cells/genotype (WT [*n* = 3], rpL24^+/–^ [*n* = 2], *P* < 0.0001, *t* test). (**B**) Kaplan-Meier survival analysis of WT (*n* = 17) and rpL24^+/–^ (*n* = 13) mice treated with 0.075% BBN ad libitum (*P* = 0.02, log-rank test). (**C**) Puromycin incorporation in normal and tumor organoids developed from WT and WT + BBN–treated mice. Representative puromycin Western blot. Quantification of *n* = 3 biological replicates (*P* = 0.02, *t* test). (**D**) Candidate gene analysis of translation regulators by Western blot using normal and BBN tumor organoids (*n* = 3 biological replicates). The same tubulin blot is used in the PI3K-AKT-mTOR pathway and integrated stress response figures. The same tubulin blot is used in the translation initiation and translation elongation figures. (**E**) eIF4E S209 phosphorylation in WT and BBN-treated C57BL/6 mice. Representative eIF4E S209 IHC. Quantification of > 5000 cells/genotype (Normal [*n* = 2], 21 weeks on BBN [*n* = 2], *P* < 0.0001, *t* test). Scale bars: 100 μm. Data are presented as mean ± SEM. See complete unedited blots in the supplemental material.

In order to determine if robust protein synthesis is necessary for bladder tumor formation, we used a mouse model haploinsufficient for the ribosomal protein L24 (rpL24^+/–^). rpL24^+/–^ mice have a pleiotropic phenotype that includes a white ventral midline, white hind feet, and a kinked tail. rpL24^+/–^ are 20% smaller than WT littermates (19). Using the puromycin incorporation assay to measure de novo protein synthesis in vivo (20), we observed that rpL24^+/–^ mice display a 60% decrease in protein synthesis within bladder urothelium, the tissue of origin for urothelial carcinoma, compared with WT mice (Figure 1A) (19, 21, 22). Importantly, we found that rpL24^+/–^ mice develop normal bladders as determined by histological analysis, demonstrating that a reduction in global protein synthesis does not impact the formation or maintenance of normal urothelium (Supplemental Figure 1; supplemental material available online with this article; https://doi.org/10.1172/jci.insight.144920DS1). Therefore, this model, which exhibits a decrease in protein synthesis, enabled us to generally decrease mRNA translation levels without affecting homeostatic functions, which is why we chose this model to study the impact of reducing protein synthesis on bladder cancer formation. We used N-butyl-N-(4-hydroxybutyl) nitrosamine (BBN), a potent bladder-tropic procarcinogen found in cigarette smoke (the most common cause of bladder cancer; ref. 23), to drive cellular transformation in WT and rpL24^+/–^ mice (24–26). Importantly, BBN treatment in mice leads exclusively to muscle-invasive urothelial carcinomas that have been shown by Fantini et al. to be similar at a genomic and histologic level to human bladder cancer (27). We found that BBN administered in the drinking water caused invasive urothelial carcinoma after just 15 weeks of treatment in WT mice. By 21 weeks, nearly all WT mice grew invasive bladder cancer characterized by hematuria (blood in the urine) and hydronephrosis (distended kidneys from obstruction), which severely compromised their survival (Supplemental Figure 2, A and B). Histologic analysis revealed that this model produced hyperplasia and invasive urothelial carcinoma (Supplemental Figure 2B). We conducted a survival study using BBN in the 2 murine cohorts and observed a significant delay in bladder cancer–related deaths in rpL24^+/–^ mice (Figure 1B). The first WT mouse died from bladder cancer after 69 days of starting BBN, while the first reported death in rpL24^+/–^ mice occurred after 140 days. Furthermore, the median survival of WT mice was 167 days compared with 191 days for rpL24^+/–^ mice (*P* = 0.02). Overall, rpL24^+/–^ mice had a 12% increase in total lifespan on BBN compared with WT mice. All mice that died had histologic evidence of bladder cancer. However, age-matched rpL24^+/–^ mice developed smaller tumors compared with WT mice (Supplemental Figure 3A). We also confirmed by mass spectrometry that rpL24^+/–^ mice concentrated high levels of BCPN — the carcinogenic metabolite of BBN (25) — in the urine, similar to WT mice (Supplemental Figure 3B). Therefore, the improvement in survival and small tumors was not a result of decreased exposure to carcinogen in rpL24^+/–^ mice. As such, robust protein synthesis is needed for efficient urothelial cancer initiation, and a decrease in protein synthesis significantly delayed the onset of death and ultimately extended the survival of mice from bladder cancer.

### Urothelial carcinoma is associated with increased protein synthesis and phosphorylation of the translation initiation factor eIF4E.

These findings demonstrate the requirement of optimal protein synthesis for bladder cancer pathogenesis and raise the question of how protein synthesis is deregulated in bladder cancer. As such, we first sought to determine how the process of cellular transformation alters de novo protein synthesis rates in the urothelium. To this end, we conducted an immunoblot-based puromycin incorporation assay to measure new protein synthesis in normal primary bladder urothelial organoids and organoids derived from BBN-transformed bladder tumors. We observed a significant 2-fold increase in new protein synthesis in the context of cancer, which we confirmed by [^35^S]-methionine incorporation (Figure 1C and Supplemental Figure 3C). This led us to ask if a specific node of the protein synthesis apparatus was associated with transformation in the BBN urothelial carcinoma model. We conducted a candidate gene analysis of critical regulators of mRNA translation previously associated with cancer formation, including: the mTOR signaling pathway, the translation initiation inhibitor eIF4E binding protein 1 (4EBP1), the translation elongation factor eEF2, the integrated stress response target eukaryotic initiation factor 2α (eIF2α), and eIF4E (11, 28–31). We measured the protein levels and activation status of each candidate by Western blot analysis of primary bladder cancer organoids developed from BBN-treated mice. From these candidates, we observed an increase in eIF2α phosphorylation, which is known to decrease global protein synthesis as a stress response (32), and well as a slight decrease in 4EBP1 phosphorylation (Figure 1D). However, we noticed that eIF4E phosphorylation at S209 was upregulated in cancerous compared with normal urothelial organoids (Figure 1D). We also found that phospho-eIF4E levels increased significantly, from normal urothelium to invasive carcinoma in vivo (Figure 1E). These findings demonstrate that the activation of a central regulator of mRNA translation initiation, eIF4E, is strongly associated with increased protein synthesis and bladder cancer.

**Figure 2:**
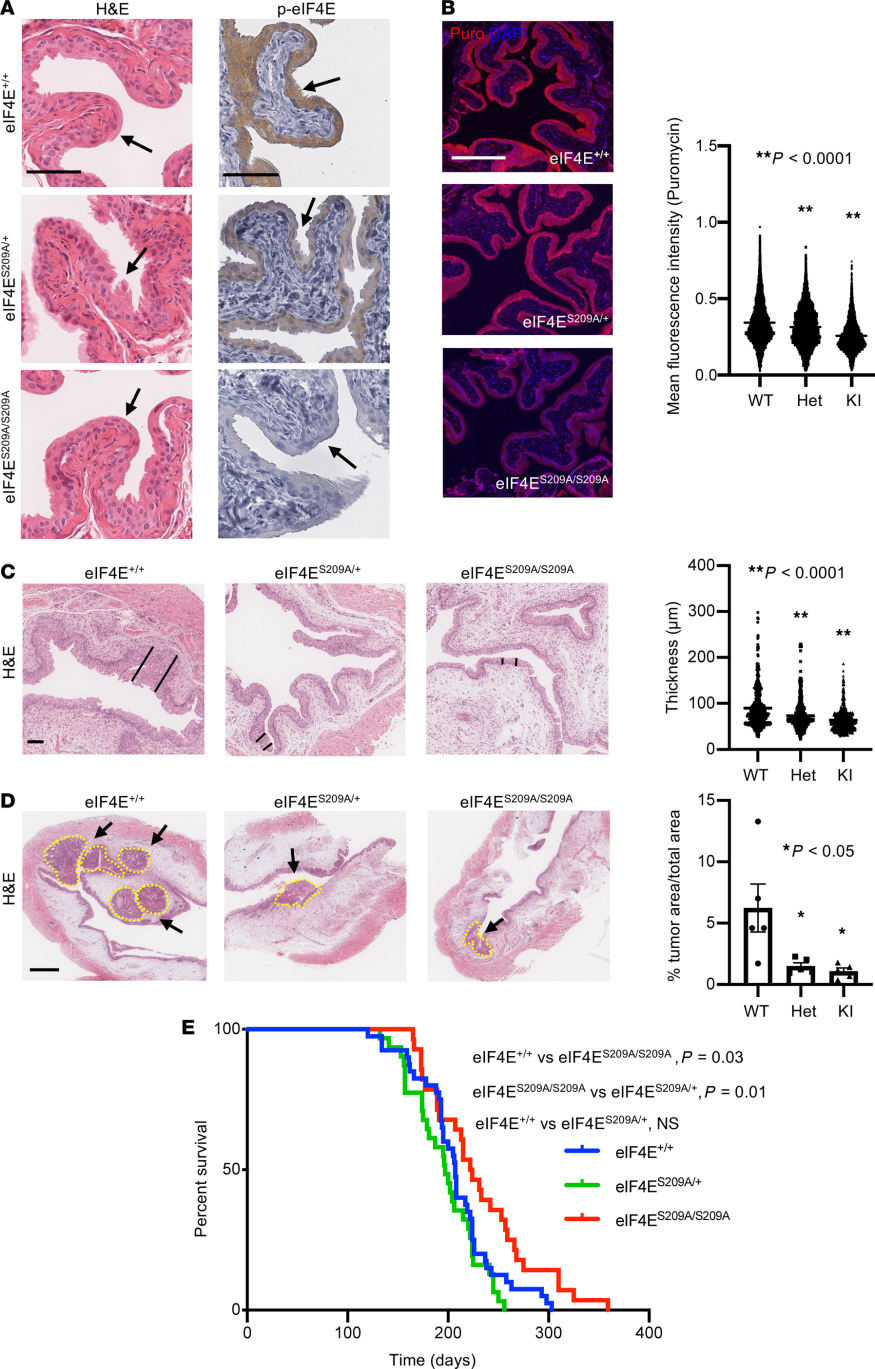
Phosphorylated eIF4E is necessary for bladder tumor initiation in vivo. (**A**) Representative H&E staining of the urothelium in eIF4E^+/+^ (WT), eIF4E^S209A/+^ (Het), and eIF4E^S209A/S209A^ (KI) mice. Representative phospho-eIF4E S209 staining in eIF4E^+/+^, eIF4E^S209A/+^ (Het), and eIF4E^S209A/S209A^ mice. (**B**) Representative puromycin IF of eIF4E^+/+^ (WT), eIF4E^S209A/+^ (Het), and eIF4E^S209A/S209A^ (KI) urothelium with quantification of > 5000 cells/genotype (*n* = 4 mice/genotype, **P < 0.0001, Dunnett’s multiple-comparison test). (**C**) Representative H&E of 9-week BBN–treated eIF4E^+/+^ (WT), eIF4E^S209A/+^ (Het), and eIF4E^S209A/S209A^ (KI) mouse urothelium used for thickness measurements (black lines demarcate urothelial thickness) with quantification (*n* = 6 mice/genotype, **P < 0.0001, Dunnett’s multiple-comparison test). (**D**) Representative H&E of 15-week BBN–treated eIF4E^+/+^ (WT), eIF4E^S209A/+^ (Het), and eIF4E^S209A/S209A^ (KI) mouse urothelium (dotted yellow lines demarcate tumors) with quantification (*n* = 6 mice/genotype, *P < 0.05 [eIF4E^+/+^ (WT) versus eIF4E^S209A/+^ (Het), *P* = 0.02; eIF4E^+/+^ (WT) versus eIF4E^S209A/S209A^ (KI), *P* = 0.01], Dunnett’s multiple-comparison test. Scale bar: 500 μm). (**E**) Kaplan-Meier survival analysis of BBN-treated eIF4E^+/+^ (*n* = 40), eIF4E^S209A/+^ (*n* = 31), and eIF4E^S209A/S209A^ (*n* = 28) mice (log-rank test). Scale bars: 100 μm unless otherwise noted. Data are presented as mean ± SEM.

*Phosphorylation of eIF4E at S209 is dispensable for normal urothelial homeostasis and necessary for carcinogen-induced bladder tumor initiation*. To further investigate the role of eIF4E phosphorylation in bladder cancer formation, we utilized the eIF4E S209 mutant mouse model (eIF4E^S209A/+^, eIF4E^S209A/S209A^) (31). In this knock-in model, the serine at position 209 has been replaced by an alanine that cannot be phosphorylated. This mouse model develops normally without any change in body size compared with WT mice (Supplemental Figure 4A). IHC analysis of the urothelium confirmed a progressive decrease in eIF4E phosphorylation comparing WT, heterozygous, and homozygous mice (Figure 2A). Furthermore, the bladder epithelium in eIF4E^S209A/+^ and eIF4E^S209A/S209A^ mice develop normally, suggesting that eIF4E phosphorylation is not required for normal bladder tissue formation and maintenance (Figure 2A). We next asked whether a single mutation to S209 was sufficient to impact de novo protein synthesis within the bladder. Using the puromycin incorporation assay, we found that eIF4E^S209A/+^ and eIF4E^S209A/S209A^ urothelium displayed decreased levels of new protein synthesis compared with WT urothelium (8.31% reduction [*P* < 0.0001] and 24.97% reduction [*P* < 0.0001], respectively) (Figure 2B). As such, eIF4E phosphorylation is necessary to maintain steady state levels of protein synthesis in the bladder urothelium but is not necessary for bladder development and homeostasis.

To understand the importance of phospho-eIF4E in bladder cancer initiation, WT, eIF4E^S209A/+^, and eIF4E^S209A/S209A^ mice were treated with BBN and all were euthanized at 9 or 15 weeks, which corresponds to the initiation of precancerous lesions and tumors, respectively. We observed that abrogating eIF4E phosphorylation significantly reduced urothelial thickness of precancerous lesions in 9-week–treated eIF4E^S209A/+^ and eIF4E^S209A/S209A^ mice compared with WT mice by 18.6% and 29.3%, respectively (Figure 2C). Moreover, we observed, after 15 weeks of BBN treatment, that WT mice had tumors approximately 5 times larger than eIF4E^S209A/+^ and eIF4E^S209A/S209A^ mice (Figure 2D). These findings demonstrate that deficient phosphorylation of eIF4E leads to reduced bladder cancer initiation, consistent with an impairment in cellular transformation. To understand the impact of this deficiency on bladder cancer survival, we treated an additional cohort of mice with BBN until the development of terminal bladder cancer, as marked by pathological confirmation of bladder tumors and > 20% weight loss after 3 consecutive measurements. Similar to our findings in the rpL24^+/–^ mouse model, eIF4E^S209A/S209A^ mice displayed a longer time to first death, as well as a significantly extended lifespan on BBN compared with WT and eIF4E^S209A/+^ mice (median survival: WT = 207 days, eIF4E^S209A/+^ = 197 days, and eIF4E^S209A/S209A^ = 223 days [*P* = 0.03 for WT versus eIF4E^S209A/S209A^; *P* = 0.01 for eIF4E^S209A/+^ versus eIF4E^S209A/S209A^]) (Figure 2E). Importantly, the 16–26 day increase in survival translates into a 10% increase in the median lifespan compared with the WT mice in this study. We also confirmed by mass spectrometry that eIF4E^S209A/S209A^ mice concentrated high levels of BCPN in the urine, similar to WT mice (Supplemental Figure 4B). Together, these findings show that phospho-eIF4E is necessary for efficient carcinogen-induced tumor initiation.

### eIF4E S209 phosphorylation is necessary for bladder cancer progression.

**Figure 3:**
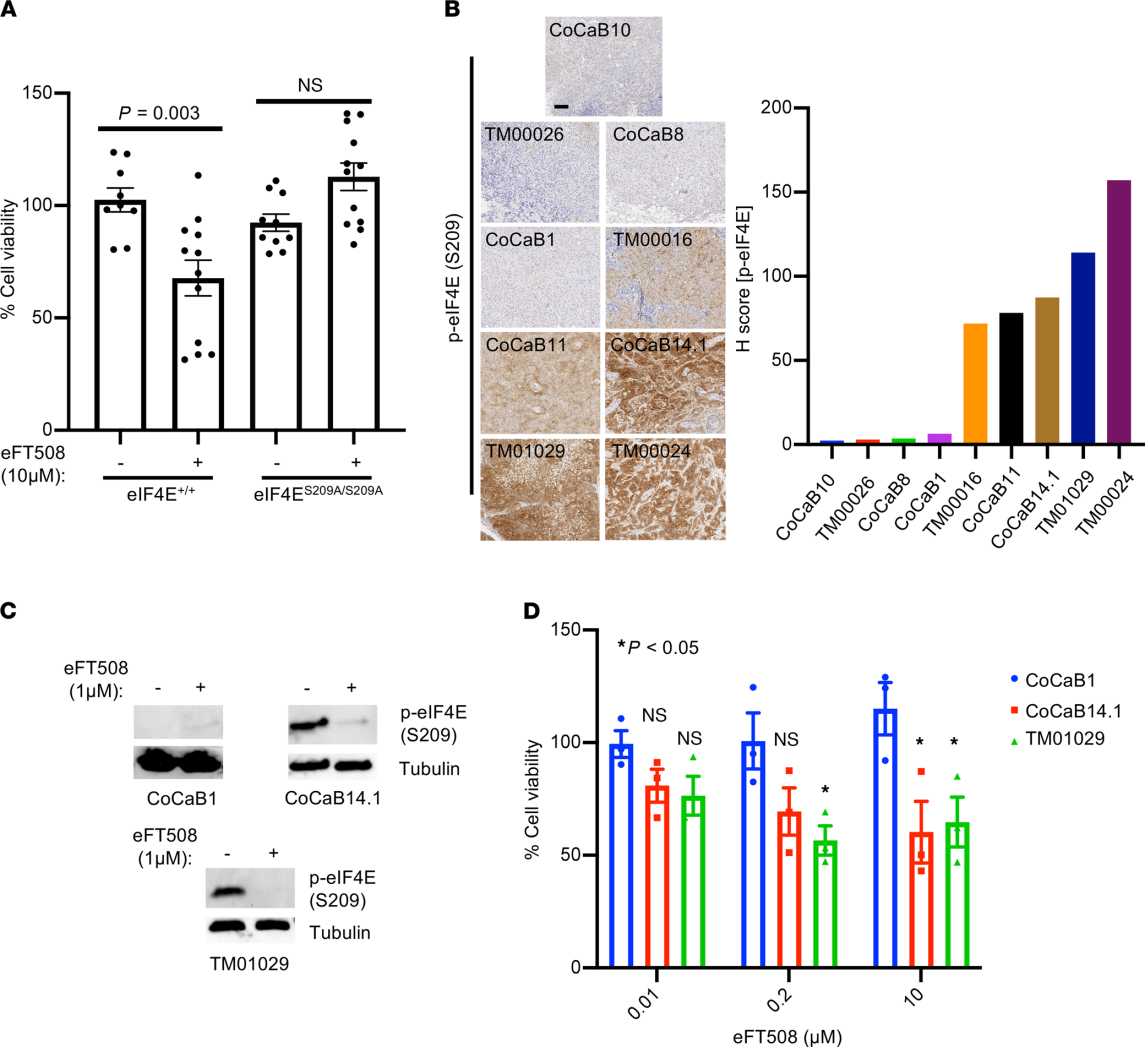
High eIF4E phosphorylation correlates with responsiveness to pharmacologic MNK1/2 inhibition in murine and human bladder cancer. (**A**) Cell viability assay in eIF4E^+/+^ and eIF4E^S209A/S209A^ bladder cancer organoids derived from BBN-treated mice (*n* = 3 biological replicates, *P* = 0.003, *t* test). (**B**) Phospho-eIF4E S209 staining across 9 bladder cancer PDX models with quantification. Scale bars: 500 μm. (**C**) Representative phospho-eIF4E Western blots across 3 bladder cancer organoid models treated with eFT508 (72 hours). (**D**) Cell viability assay of the CoCaB1, CoCaB14.1, and TM01029 PDX derived organoids treated with 0.01, 0.2, or 10 μM eFT508 for 72 hours. *P < 0.05 (CoCaB1 versus TM01029 at 0.2 μM, *P* = 0.03; CoCaB1 versus CoCaB14.1 at 10 μM, *P* = 0.03; CoCaB1 versus TM01029 at 10 μM, *P* = 0.04). Dunnett’s multiple-comparison test. Scale bar: 100 μm. Data are presented as mean ± SEM.

Given our observation that eIF4E phosphorylation is necessary for efficient bladder cancer formation (Figure 2, C–E), we next asked whether eIF4E phosphorylation is required for the maintenance of established tumors. The primary kinases responsible for the phosphorylation of eIF4E are MNK1 and MNK2 (17, 33). It has also been shown that both kinases are necessary for PTEN loss–mediated tumorigenesis (34). Furthermore, a potent and highly selective MNK1 and MNK2 inhibitor called eFT508 was recently developed and is currently in clinical trials for patients with advanced cancers (14). To determine if eIF4E phosphorylation is necessary for bladder cancer progression, we treated WT primary bladder cancer organoids with eFT508. We observed that eIF4E phosphorylation–competent tumors are sensitive to the inhibitory effects of MNK1 and MNK2 inhibition (Figure 3A). This finding shows that MNK1 and MNK2 activity are critical for the growth of established bladder cancer. However, it raises the question of whether eIF4E is the substrate responsible for the therapeutic response to eFT508. To this end, we generated eIF4E^S209A/S209A^ bladder cancer organoids from BBN-induced bladder cancer mice and treated them with eFT508. Remarkably, eIF4E phosphorylation–defective mutant organoids were completely impervious to the antitumor effects of the drug (Figure 3A). These findings show that the growth inhibitory effects of eFT508 are mediated by the on-target inhibition of the MNK1/2-eIF4E signaling pathway. Thus, eIF4E phosphorylation is required for bladder cancer progression and represents a potential biomarker for responsiveness to MNK1 and MNK2 inhibition in urothelial malignancies.

### eIF4E S209 phosphorylation is a requisite for a therapeutic response to eFT508 in bladder cancer.

To determine if these findings can be extended to human models of urothelial cancer, we screened a series of bladder cancer PDX models. We observed that 5 of 9 PDX lines exhibited moderate to high levels of eIF4E S209 phosphorylation, while 4 exhibited negligible levels of phosphorylation (Figure 3B). This finding demonstrates the heterogeneity of eIF4E S209 phosphorylation across models of bladder cancer. We subsequently created organoid models from 1 low eIF4E phosphorylation model (CoCaB1) (35) and 2 high eIF4E phosphorylation models (CoCaB14.1 and TM01029), which recapitulated the in vivo models (Supplemental Figure 5). These low-passage organoid lines were treated with increasing concentrations of eFT508 (0.01–10 μM), validated for target inhibition (Figure 3C), and measured for cell viability. Both high phospho-eIF4E lines (CoCaB14.1 and TM01029) demonstrated a dose-dependent decrease in cell viability in response to eFT508 (Figure 3D). Importantly, eFT508 did not have any cytotoxic effects on the phospho-eIF4E–negative line (CoCaB1) at all concentrations tested (Figure 3D). Taken together, our data demonstrate that phospho-eIF4E levels in human bladder cancer organoids positively correlate with responsiveness to MNK1 and MNK2 inhibition.

**Figure 4:**
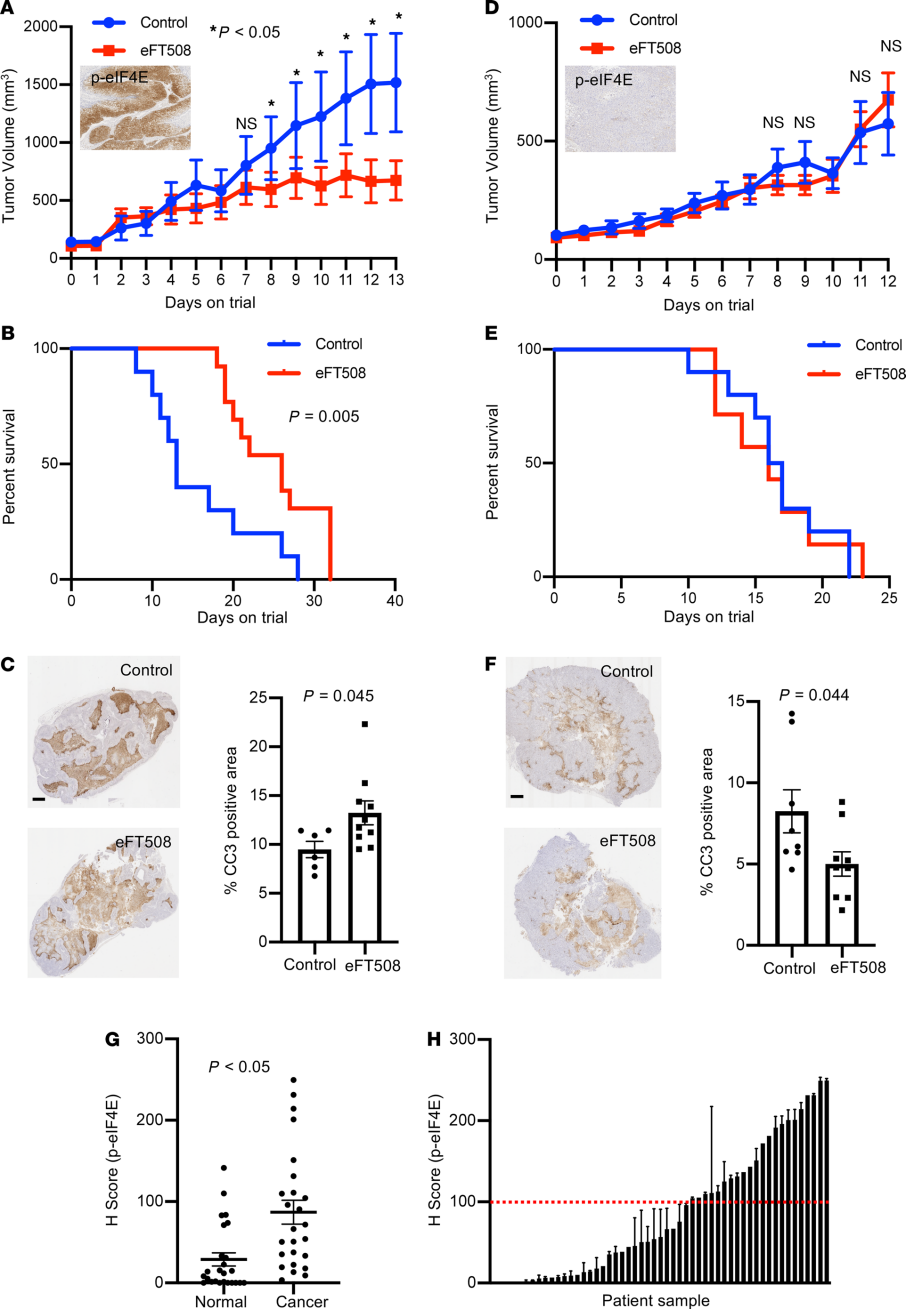
eFT508 treatment causes reduced tumor size and improved survival in phospho-eIF4E high bladder cancer PDX model. (**A**–**C**) Growth curve (Inset*:* representative phospho-eIF4E staining in the TM01029 PDX) (**A**), Kaplan-Meier curve (**B**), and representative IHC and bar graph of cleaved caspase 3 (CC3) (**C**) of the TM01029 (high phospho-eIF4E) PDX model treated daily with eFT508 10 mg/kg orally (*n* = 10 [control]; *n* = 13 [eFT50])). (**D**–**F**) Growth curve (*Inset:* representative phospho-eIF4E staining in the CoCaB1 PDX) (**D** Kaplan-Meier curve (**E**), and representative IHC and bar graph of cleaved caspase 3 (CC3) (**F**) of the CoCaB1 (low phospho-eIF4E) PDX model treated daily with eFT508 10 mg/kg orally (*n* = 9 [control]; *n* = 8 [eFT508]). (**G**) Phospho-eIF4E S209 levels in primary bladder cancer specimens compared with matched normal tissues (*n* = 25 patients, *P* < 0.05, *t* test). (**H**) Phospho-eIF4E S209 levels across 53 primary bladder cancer specimens demonstrating that 37% of patients express high levels of phosphorylated eIF4E. Scale bars: 1 mm. Data are presented as mean ± SEM.

Next, we sought to determine if these in vitro finding were applicable to in vivo models of human bladder cancer. To this end, we used the CoCaB1 (phospho-eIF4E low) and TM01029 (phospho-eIF4E high) PDX models (Figure 3B). Mice were randomized to either 10 mg/kg of eFT508 or vehicle daily by oral gavage. We observed that the phospho-eIF4E high line was exquisitely sensitive to MNK1 and MNK2 inhibition, which led to significantly smaller tumors and an improvement in survival (Figure 4, A and B). At a cellular level, we observed that eFT508 treatment led to an increase in apoptosis (Figure 4C). Mice that received eFT508 exhibited no overt toxicity (Supplemental Figure 6). Remarkably, the phospho-eIF4E low line was completely insensitive to eFT508, with both the vehicle and drug treatment groups perishing at the same rate with no observable change in apoptosis (Figure 4, D–F). These results show that eFT508 functions through the precise inhibition of the MNK1/2-eIF4E signaling pathway. Furthermore, phospho-eIF4E plays an important role in bladder cancer progression, and targeting its upstream kinases could be an effective therapeutic option for bladder cancer patients with elevated phospho-eIF4E.

To determine the number of muscle invasive bladder cancer patients who could potentially benefit from an inhibitor of eIF4E phosphorylation, we conducted phospho-eIF4E S209 IHC of human urothelial carcinoma tissue specimens. For those with matched normal tissues (*n* = 25), we observed a significant increase in eIF4E phosphorylation in the setting of cancer compared with noncancerous urothelium as we have observed in WT BBN-treated mice (Figure 4G and Figure 1E). Moreover, we found that approximately 37% of patients with muscle-invasive disease expressed high levels of phospho-eIF4E (Figure 4H). As such, it is possible that more than one-third of patients with advanced stage bladder cancer may be good candidates for a therapeutic trial of an eIF4E phosphorylation inhibitor such as eFT508.

## Discussion

Protein synthesis represents a fundamental molecular process that is critical for cancer behavior (36). However, not all types of cancers share the same dependencies on protein synthesis. For example, it has been shown that, while MYC-induced lymphomas are sensitive to decreases in protein synthesis, p53-induced solid tumors are impervious to similar decreases (37). Moreover, not all aspects of the protein synthesis machinery, even when they are converged upon by the same upstream pathway, are required for tumor initiation and progression. It has been shown that, despite the fact that mTOR regulates protein synthesis through posttranslational control of the 4EBP1/eIF4E and the S6K/rpS6 axes, the former and not the latter is required for AKT-mediated tumorigenesis of the thymus (28). However, in a genetically engineered model of pancreatic insulinoma, the S6K-rpS6 pathway is essential for tumor development (38). As such, different tissue types have varying quantitative and qualitative requirements for protein synthesis to drive the process of tumor formation. Therefore, in order to understand whether a specific tissue type requires aberrant protein synthesis for the development of tumors, it is imperative to evaluate the tissue of origin in question.

Using a confluence of carcinogen-based genetically engineered mouse models, we demonstrate that the bladder urothelium requires robust protein synthesis to promote the process of cellular transformation and tumor growth. This is mediated, in part, through hyperphosphorylation of the oncogene eIF4E, since genetic perturbation of this posttranslational modification delays tumor initiation and progression, and improves overall survival. Moreover, we found that eIF4E phosphorylation levels dictate the ability of bladder tumors to respond to the clinical-grade MNK1 and MNK2 inhibitor eFT508. Importantly, this work demonstrates that the functional target of eFT508 in bladder cancer is eIF4E and that inhibition of its phosphorylation is required for its therapeutic efficacy. Moreover, these findings show that inhibition of eIF4E phosphorylation does not inhibit the growth of all tumors indiscriminately but, rather, requires a state of oncogenic addiction to this prominent posttranslational modification. Importantly, eIF4E phosphorylation has been demonstrated to be important in other cancers, including malignancies of the breast, colon, and prostate (31, 39, 40). Currently, phase 2 clinical trials are being conducted using eFT508 in combination with checkpoint inhibitors and concurrently with chemotherapy in metastatic triple-negative breast cancer (NCT03616834 [https://clinicaltrials.gov/ct2/show/NCT03616834?term=NCT+03616834&draw=2&rank=1] and NCT04261218 [https://clinicaltrials.gov/ct2/show/NCT04261218?term=NCT+04261218&draw=2&rank=1]). This study and the prevalence of eIF4E hyperphosphorylation within muscle-invasive bladder cancer provide the preclinical rationale for conducting phase 2 studies in urothelial carcinoma patients.

## Methods

### Mice.

eIF4E^S209A/S209A^ mice (a gift from Nahum Sonenberg, McGill University, Montreal, Canada) were mated with C57BL/6 mice to obtain the eIF4E^S209A/+^ line. All mice were maintained in a C57BL/6 background. Mice were genotyped using polymerase chain reaction using primers (4EKI reverse, 5’-GCAATGCAAGTCGAAATGTG-3’; 4EKI forward, 5’-TTTGAAATTGGTTTGTAAAGTTGG-3’). rpL24^+/–^ mice were obtained from the Jackson Laboratory. For all BBN experiments, BBN was administrated (0.075%) in drinking water ad libitum. Mice were euthanized based on > 20% weight loss from baseline or presence of blood in urine.

### Urine BCPN analysis.

Urine was collected from either WT and rpL24^+/–^ mice or WT and eIF4E^S209A/S209A^ mice after 9 days of 0.075% BBN treatment. A total of 5 uL of urine was analyzed on a Xevo QTof mass spectrometer. BCPN concentrations were calculated by comparing the urine BCPN measurements to standards of known BCPN concentrations also measured by mass spectrometry.

### Organoid culture.

Organoid lines were generated either from normal tissue or bladder tumor samples. Minced tissue was treated with collagenase solution (5 mg/mL) for 1 hour at 37°C, followed by a 5-minute TrypLE treatment at 37°C. Digested tissue was dissociated using a 18G syringe, and single cells were obtained after passing through a cell strainer. Cells were plated using Matrigel and incubated in organoid media for 7–10 days at 37°C. Organoids were passaged once every week. Bladder cancer organoids were cultured in ADMEM/F12 medium supplemented with 20% B27 (Thermo Fisher Scientific, 17504-044), 10 mM HEPES, Glutamax (Thermo Fisher Scientific, 35050061), and 1.25 mM N-Acetyl-L-cysteine (Sigma-Aldrich, A9165). Base media was also supplemented with 50 ng/mL EGF (Peprotech, 315-09), 100 ng/mL Noggin (conditioned media), 500 ng/mL R-spondin (conditioned media), 200 nM A83-01 (Tocris, 2939), and 10 μM Y-27632 (Sigma-Aldrich, Y0503).

### Reagents and antibodies.

eFT508 was acquired from eFFECTOR Therapeutics. BBN and BCPN were purchased from TCI Chemicals. Antibodies used were against phospho-eIF4E S209 (Abcam, ab76256), eIF4E (Santa Cruz Biotechnology Inc., sc-271480), rpS6 (Cell Signaling Technology, 2217), p-rpS6 (S240/244) (Cell Signaling Technology, 5364), p-rpS6 (S235/236) (Cell Signaling Technology, 4858), 4EBP1 (Cell Signaling Technology, 9644), p-4EBP1 (T37/46) (Cell Signaling Technology, 2855), eIF2α (Cell Signaling Technology, 2103), p-eIF2α (S51) (Cell Signaling Technology, 3597), eEF2 (Cell Signaling Technology, 2332), p-eEF2 (T56) (Cell Signaling Technology, 2331), puromycin (EMD Millipore, MABE343), and tubulin (MilliporeSigma, T8203).

### Western blot analysis.

Organoids were harvested and lysed in ice-cold RIPA buffer (Thermo Fisher Scientific, PI89900) containing protease inhibitor cocktail (Sigma-Aldrich, 11836153001) and phosphatase inhibitor (Sigma-Aldrich, 4906845001). The lysates were incubated on ice for 30 minutes, vortexed, and centrifuged at 13000 *g* at 4º C for 10 minutes. The supernatants were subjected to SDS-PAGE and were then transferred to a PDVF membrane, which was blocked with 5% milk for 1 hour at room temperature. The blot was incubated with primary antibodies in 5% milk overnight at 4°C. After 3 washes with PBS, the blot was incubated with HRP-conjugated secondary antibodies (rabbit anti–goat IgG [Thermo Fisher Scientific, A11034] or mouse anti–goat IgG [Thermo Fisher Scientific, A11032]) in 5% milk for 1 hour at room temperature. After 3 washes with PBS, West Pico (Thermo Fisher Scientific, PI34080) was used to detect immunoreactive bands on the blot using the ChemiDoc Touch Imaging System (Bio-Rad).

### Tissue histology, IHC, and immunofluorescence (IF).

Bladder samples were fixed in 4% PFA, dehydrated with ethanol, and embedded in paraffin. Sections (5 μm) were cut on a rotary microtome and stained with H&E. Immunostaining was performed on paraffin-embedded bladder tissue. In brief, the paraffin blocks were sectioned and placed on glass slides, deparaffinized with xylene, and rehydrated with decreasing concentrations of ethanol in water. Antigen retrieval was achieved by preheating sodium citrate buffer (pH 6.0) for 7 minutes at 125°C in a pressure cooker, followed by cooling at room temperature. Endogenous peroxidases were quenched by incubating the slides in hydrogen peroxide (Vector Laboratories, SP-6000) for 5 minutes. Tissue was blocked in 1% BSA. Primary antibodies were applied for 60 minutes at room temperature in a humidified chamber, and secondary antibodies (Envision system HRP-labeled polymer, Dako K4003) were applied for 60 minutes at room temperature. Color development was achieved by applying DAB solution (Agilent Technology, K3467) for 2–5 minutes, depending on the primary antibody. After washing in distilled water, the sections were counterstained with hematoxylin (Agilent technology, S3309), washed, and cover-slipped using aqueous-based mounting medium (Agilent Technology, S302580). Slides were scanned using an Aperio Scanscope AT (Leica Biosystems), and H-score calculations were made using semiautomated image analysis software (HALO, Indica Labs).

For IF detection, antigen retrieval was achieved by preheating sodium citrate buffer (pH 6.0) for 30 minutes at 95°C in a pressure cooker, followed by cooling at room temperature. Tissue was blocked using M.O.M blocking solution (MKB 2213, Vector Laboratories) for 1 hour at room temperature, followed by blocking in 1% BSA solution for 2 hours at room temperature. Primary antibodies were applied over night at 4°C in a humidified chamber, and secondary antibodies (Alexa-Fluor 594, Invitrogen) were applied for 60 minutes at room temperature. All IF slides were mounted using ProLong gold mounting media with DAPI (Invitrogen). IF slides were scanned using an Aperio Scanscope FL (Leica Biosystems), and calculations were done using semiautomated image analysis software (HALO, Indica Labs).

### In vitro and In vivo puromycin incorporation assay.

A total of 10,000 cells was plated in Matrigel (NIH) and immersed in organoid media (see organoid culture). Cells were treated with 1 μM puromycin (Thermo Fisher Scientific, BP2956100) for 30 minutes at 37°C. Cells were subjected to Western blot analysis. For in vivo puromycin incorporation assay, mice were injected i.p. with 200 μL of 2.5 mM puromycin and euthanized after 1 hour.

### In vitro [^35^S]-methionine labeling assay.

A total of 10,000 cells was plated in Matrigel (NIH) and immersed in organoid media (see organoid culture). Cells were treated with 20 mCi [^35^S]-methionine (Perkin Elmer, NEG772002MC) for 1 hour at 37°C. Cells were collected and subjected to Western blot analysis. Radioactivity was detected using x-ray film.

### Cell viability assay.

Cells were plated in 96-well plates at a density of 1000 cells per well using 5 μL of matrigel (NIH) and treated with eFT508 or DMSO for 72 hours. Organoids were disintegrated and lysed using 80 μL of TrypLE for 30 minutes at 37°C. A total of 80 μL of 2X CellTiter-Glo reagents (Thermo Fisher Scientific) was added to activate the luminescence. Luminescence was measured using a Synergy 2 multidetection Microplate Reader (BioTek). The percentage of cell death was calculated by using the manufacturer protocol (CellTiter-Glo 2.0 Assay, Thermo Fisher Scientific, PRG9242).

### Preclinical trials.

Tumor chunks (1 × 1 × 1 mm) were implanted into the flank of NOD-SCID γ IL-2 mice. Tumor volume was calculated using the formula (L[W2])/2, where L is the length of the tumor and W is the width. When tumors reached 100 mm^3^, animals were randomized to receive eFT508 (10 mg/kg per day, once, orally) or vehicle (1 Methyl-2-pyrrolidinone, anhydrous (Sigma; catalog 328634] [10% v/v] and Propylene Glycol [USP/FCC] [Fisher, catalog P355] [90% v/v]), from Monday to Sunday.

### Statistics.

All statistical analysis was performed using GraphPad Prism software (version 8.2.1). The log-rank test was used for animal survival, unpaired 2-tailed Student’s *t* test (except Figure 4A, which is 1-tailed) was used for 2-group comparisons, and a Dunnett’s multiple-comparison test was used for comparisons of more than 2 groups. Data are presented as mean ± SEM. A *P* value of less than 0.05 was considered significant.

### Study approval.

All animal procedures were performed according to animal care guidelines approved by the Institutional Animal Care and Use Committee (IACUC) at the Fred Hutchinson Cancer Research Center.

## Author contributions

SJ and ACH conceived and designed experiments. SJ, RD, and YL performed experimental studies. RH and JH acquired data. ACH and SJ wrote the manuscript. JLW, ML, KW, GC, and NS provided reagents. All authors edited the manuscript.

## Supplementary Material

Supplemental data

## Version 1 05/25/2021

In-Press Preview

## Version 2 06/08/2021

Electronic publication
