# Supplementary material for: mRNA translation is a therapeutic vulnerability necessary for bladder epithelial transformation
Source: JCI Insight. 2021 Jun 8;6(11):e144920. doi: 10.1172/jci.insight.144920 (PMC8262354; doi:10.1172/jci.insight.144920)

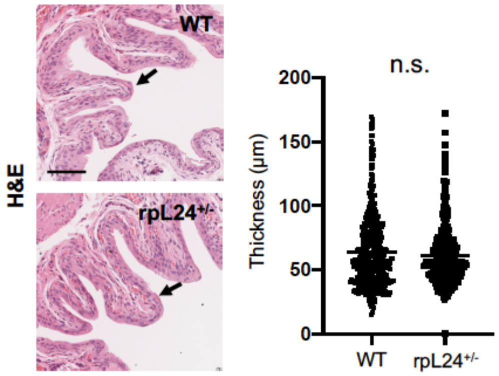


**Figure S1. rpL24+/- bladders are histologically normal.** Representative H&E staining of WT and rpL24+/- bladder urothelium (scale bar: 100 µm) with quantification (right panel, n = 3 mice/genotype, n.s. = not statistically significant).

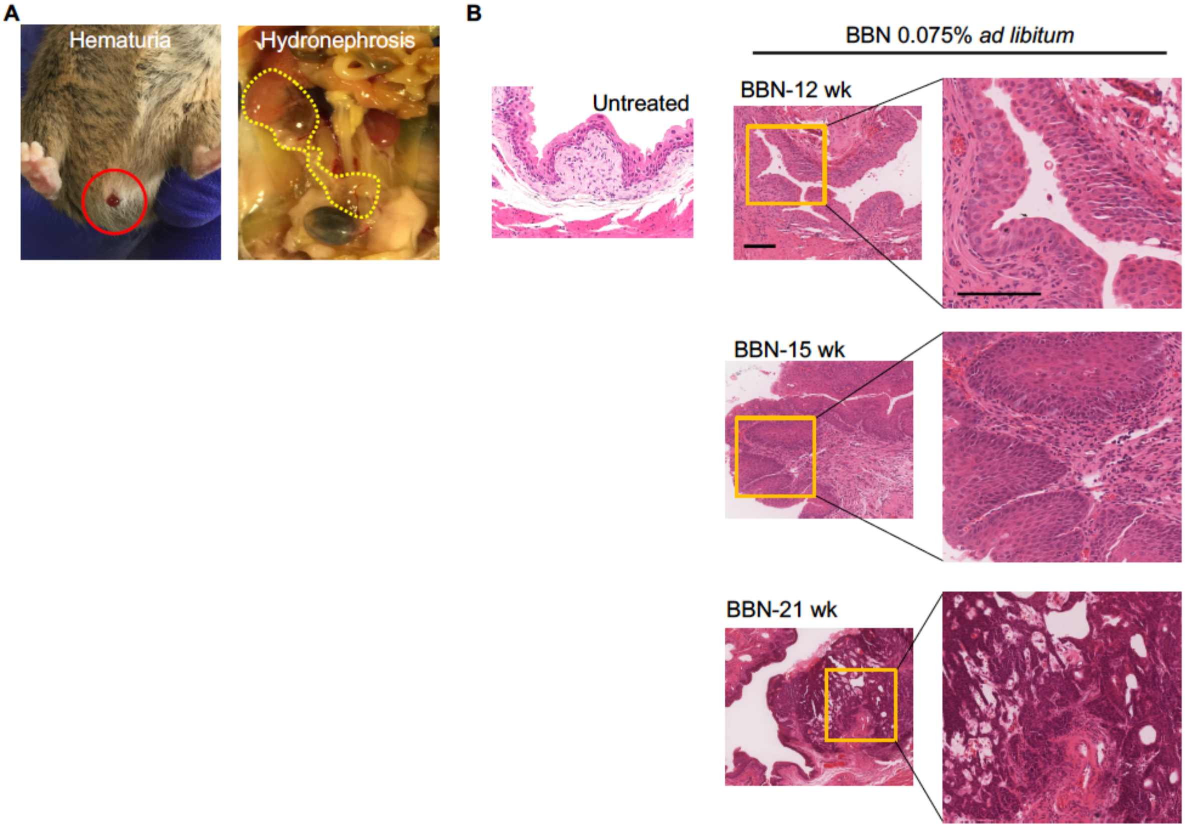


**Figure S2. Gross and microscopic effects of BBN treatment in WT mice. (A)** Representative gross images of mice treated with BBN of over 21 weeks that develop hematuria (demarcated by a red circle) and hydronephrosis (demarcated by a dotted yellow line). **(B)** H&E staining showing development of BBN-induced invasive bladder cancer in WT C57BL/6 mice (representative areas are shown at high magnification for clarity). All scale bars: 100 μm.

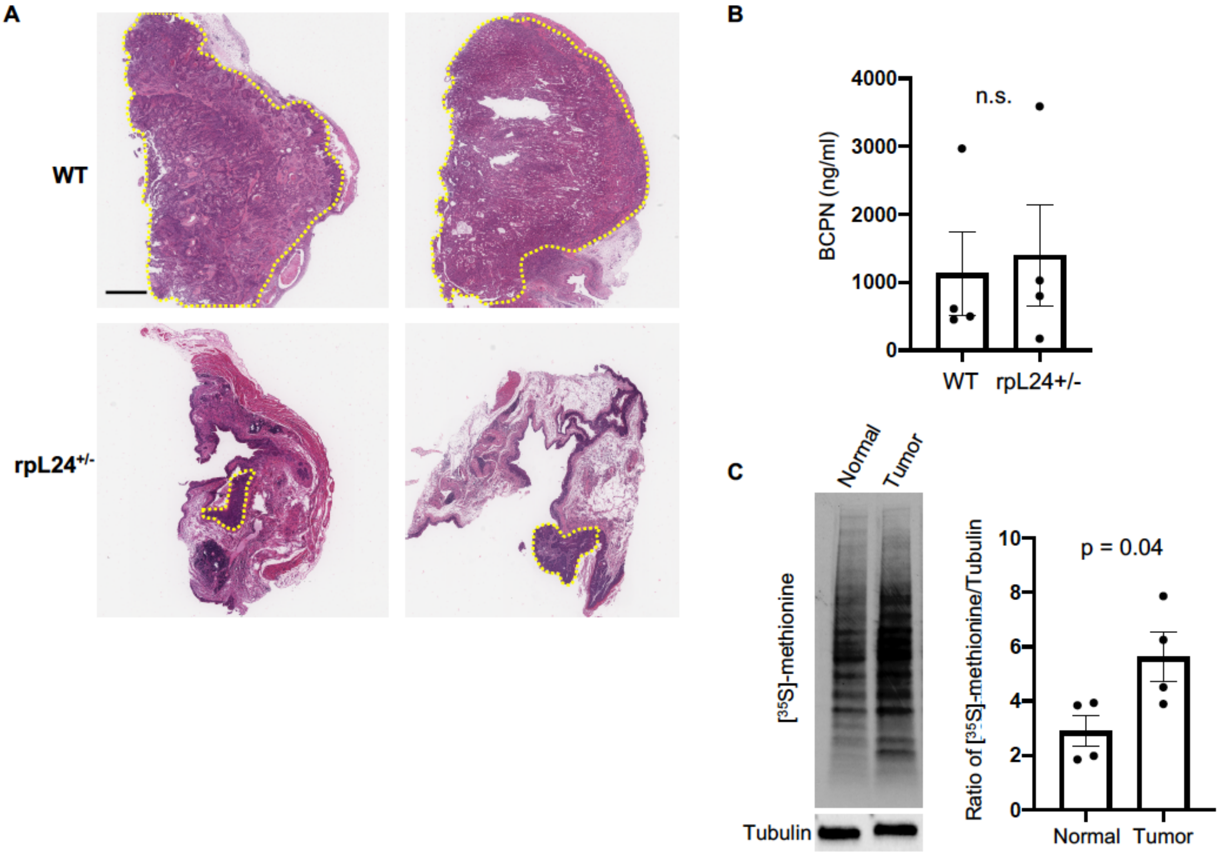


**Figure S3. rpL24+/- mice exhibit smaller bladder tumors compared to WT mice but concentrate the carcinogen BCPN in urine at levels similar to WT mice and [$^{35}$S]-methionine incorporation in WT and BBN-induced tumor organoids. (A)** Representative H&E staining showing tumor area of BBN-induced invasive bladder cancer in age-matched WT and rpL24$^{+/-}$ mice (all mice depicted were treated with BBN for 200 days, dotted yellow lines demarcate the bladder tumor, scale bar: 1 mm). **(B)** Bar graph representing mass spectrometry data of BCPN, the carcinogenic BBN metabolite, within the urine of WT and rpL24+/- mice (n = 4 mice/genotype). n.s. = not statistically significant. Data are presented as means +/- SEM**. (C)** [$^{35}$S]-methionine incorporation in WT and BBN-induced tumor organoids. *Left panel*: representative western blot. *Right panel*: quantification of n = 4 biological replicates (p = 0.04 , t-test). Data are presented as means +/- SEM**.**

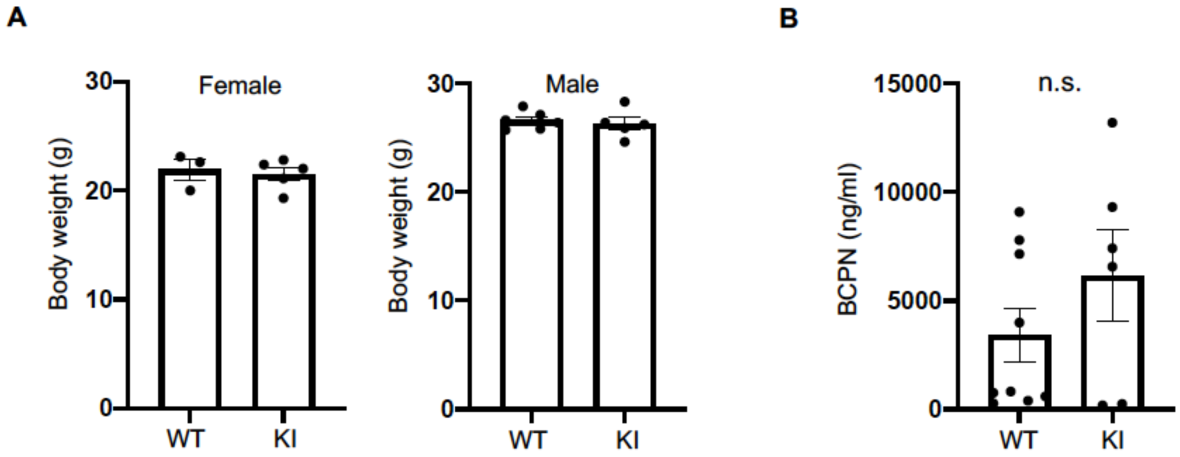


**Figure S4. eIF4E$^{S209A/S209A}$ mouse weights and urine BCPN levels. (A)** Body weight measurements of age-matched eIF4E$^{S209A/S209A}$ mice compared to WT mice. **(B)** WT and eIF4E$^{S209A/S209A}$ urine BCPN levels as determined by mass spectrometry (n = 6-9 mice/genotype). n.s. = not statistically significant. Data are presented as means +/- SEM**.**

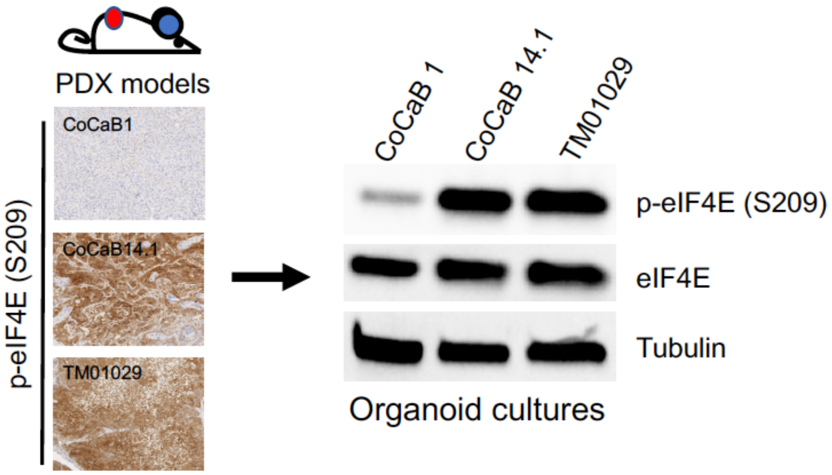


**Figure S5. Comparison of IHC and western blot analysis of PDX models.** PDX-derived primary tumor organoids reflect the phospho-eIF4E levels of the PDX models they are derived from. Comparison of eIF4E S209 phosphorylation levels between the CoCaB1, CoCaB14.1, and TM01029 PDX models and their respective organoids.

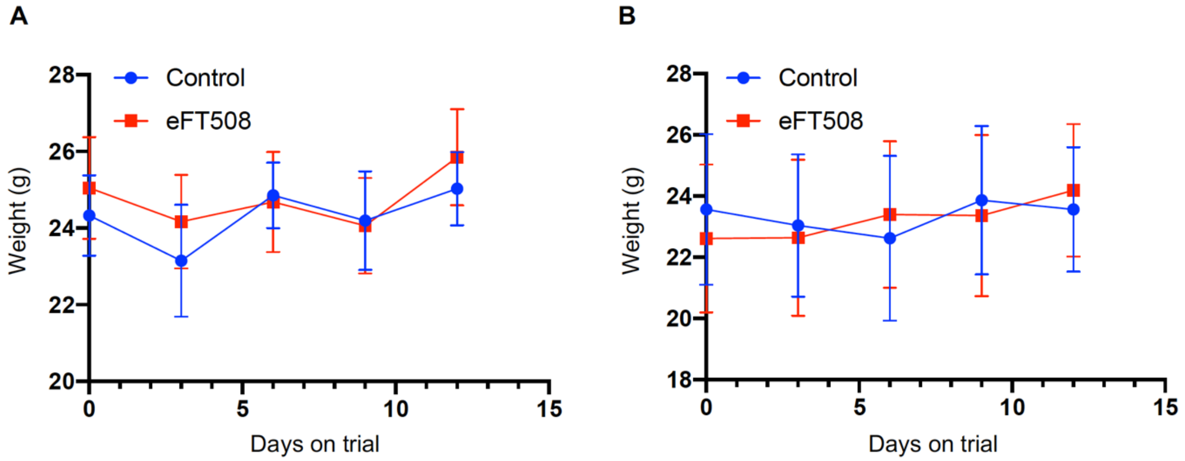


**Figure S6. eFT508 treatment is well tolerated in PDX models.** Weights of the TM01029 **(A)** and CoCaB1 **(B)** PDX models treated with eFT508 10mg/kg PO daily.

# Figure 1C

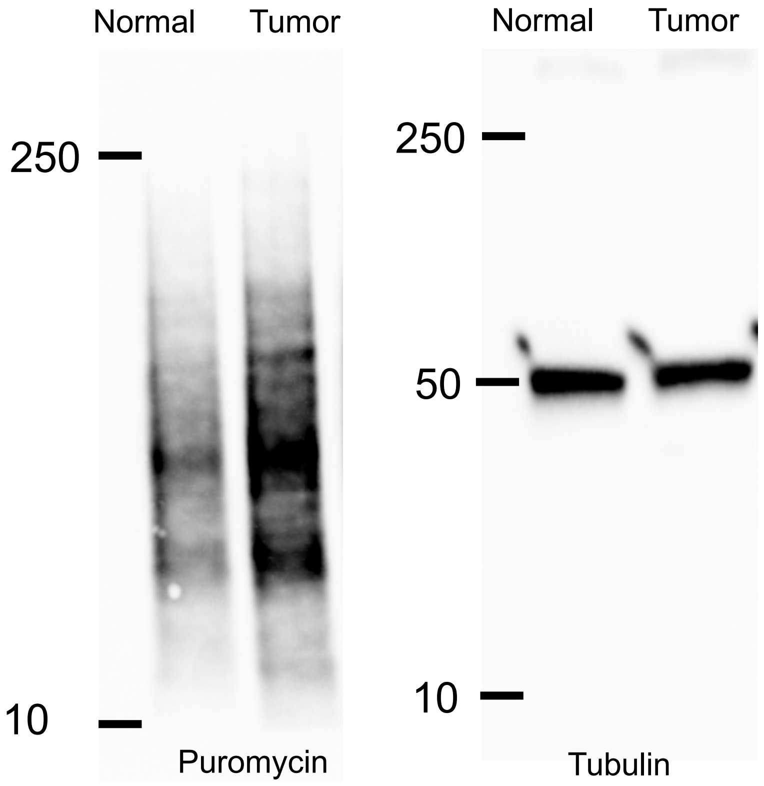
Normal
Tumor
250
10
Puromycin
Normal
Tumor
250
50
10
Tubulin

**Figure 1D**

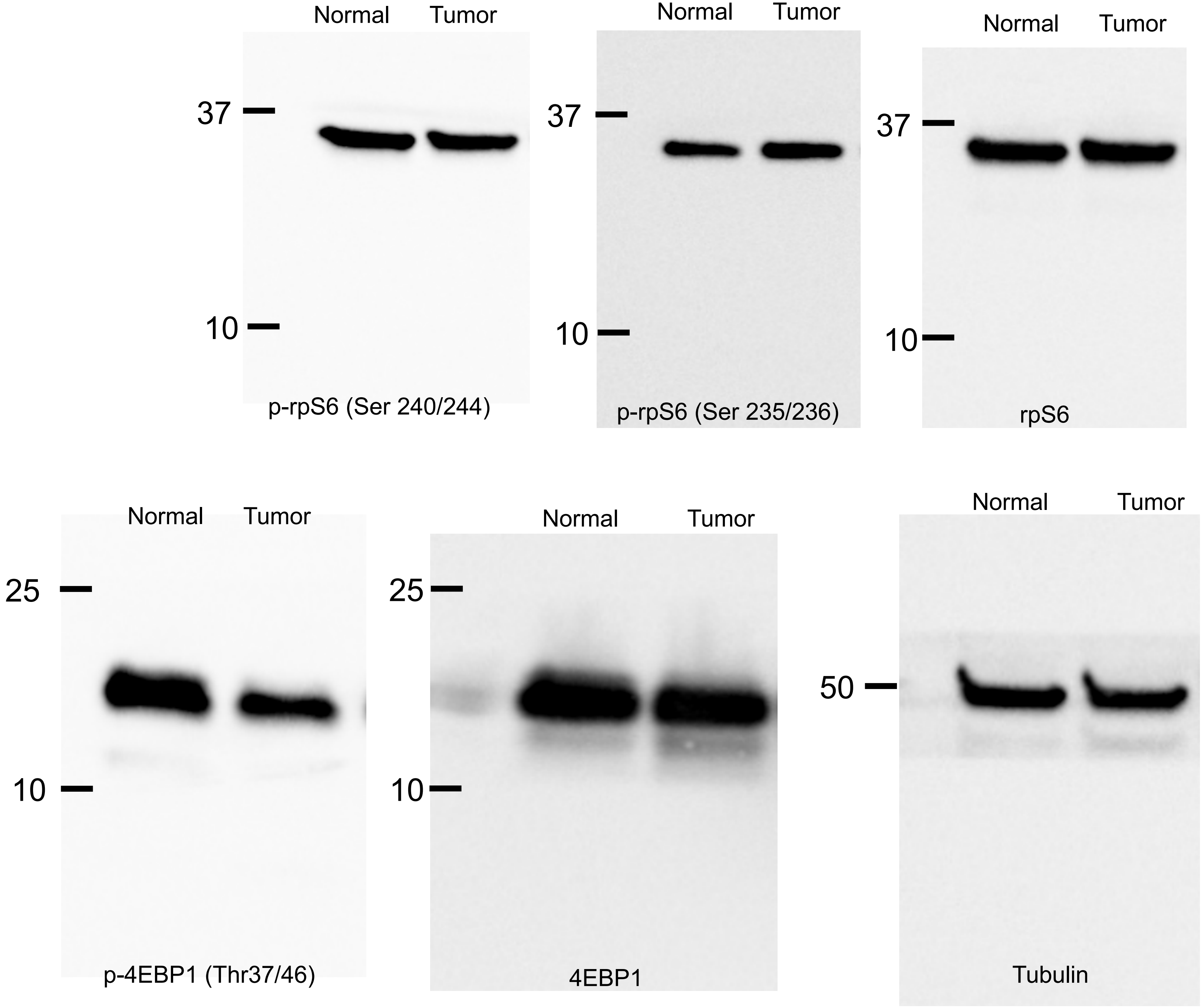


This tubulin blot was the loading control for all PI3K pathway and integrated stress response pathway blots.

## Figure 1D

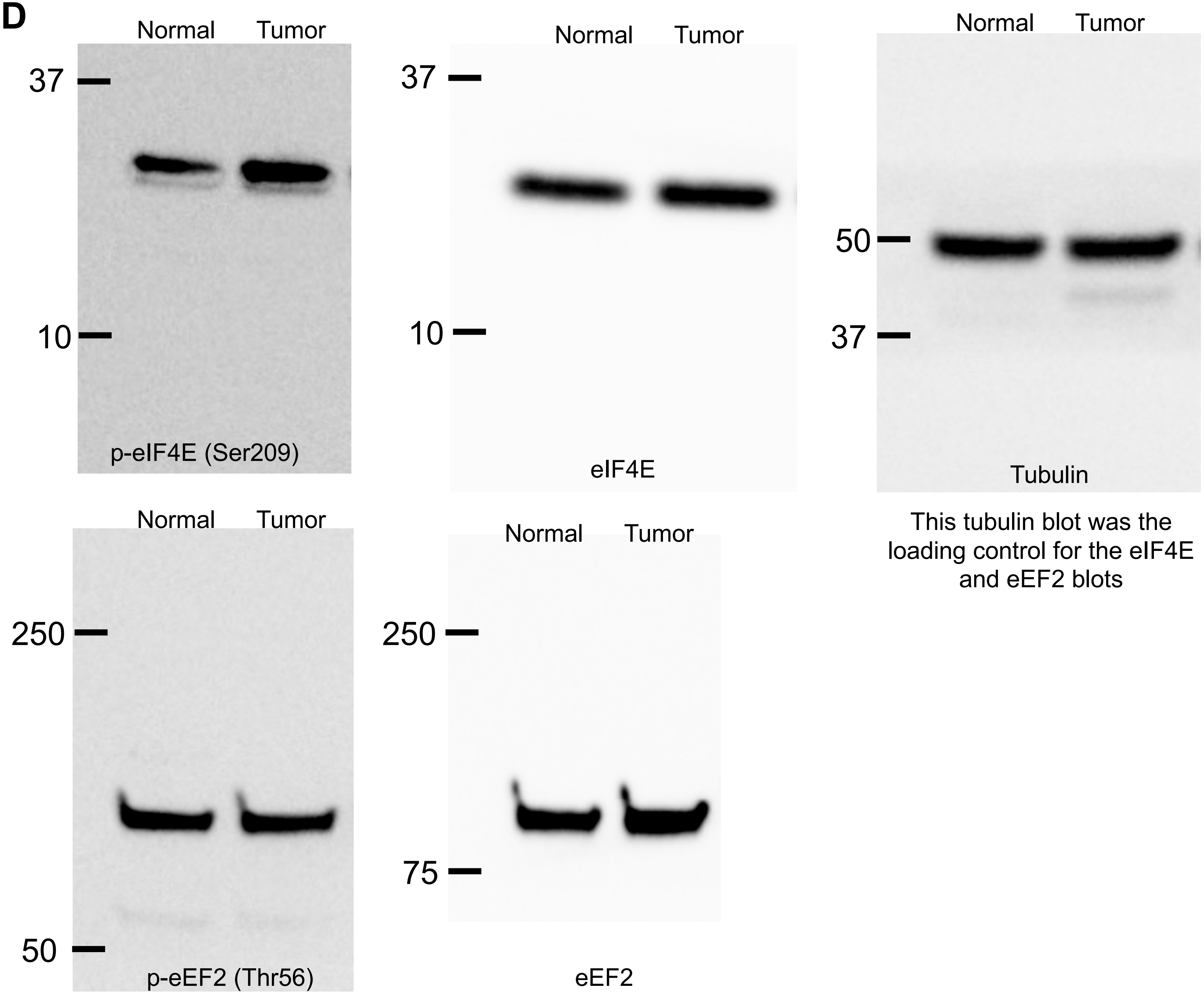


This tubulin blot was the loading control for the eIF4E and eEF2 blots

# Figure 1D

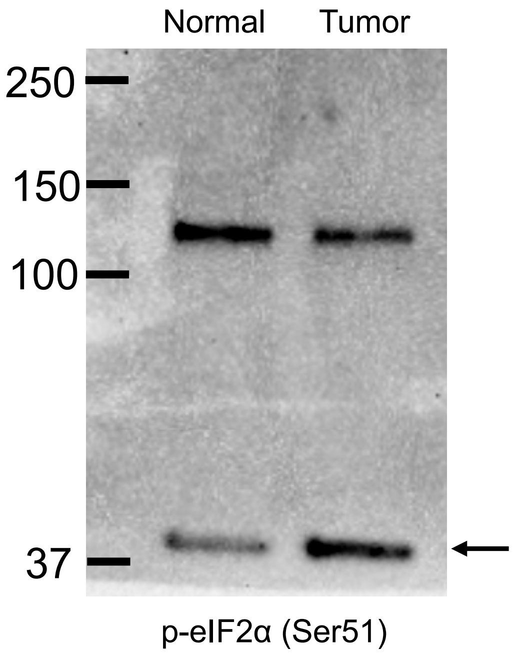
Normal
Tumor
250
150
100
37
p-eIF2α (Ser51)

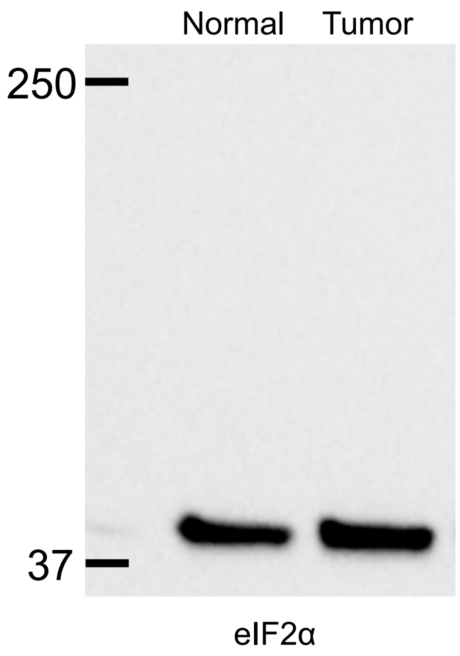
Normal
Tumor
250
37
eIF2α

# Figure 3C

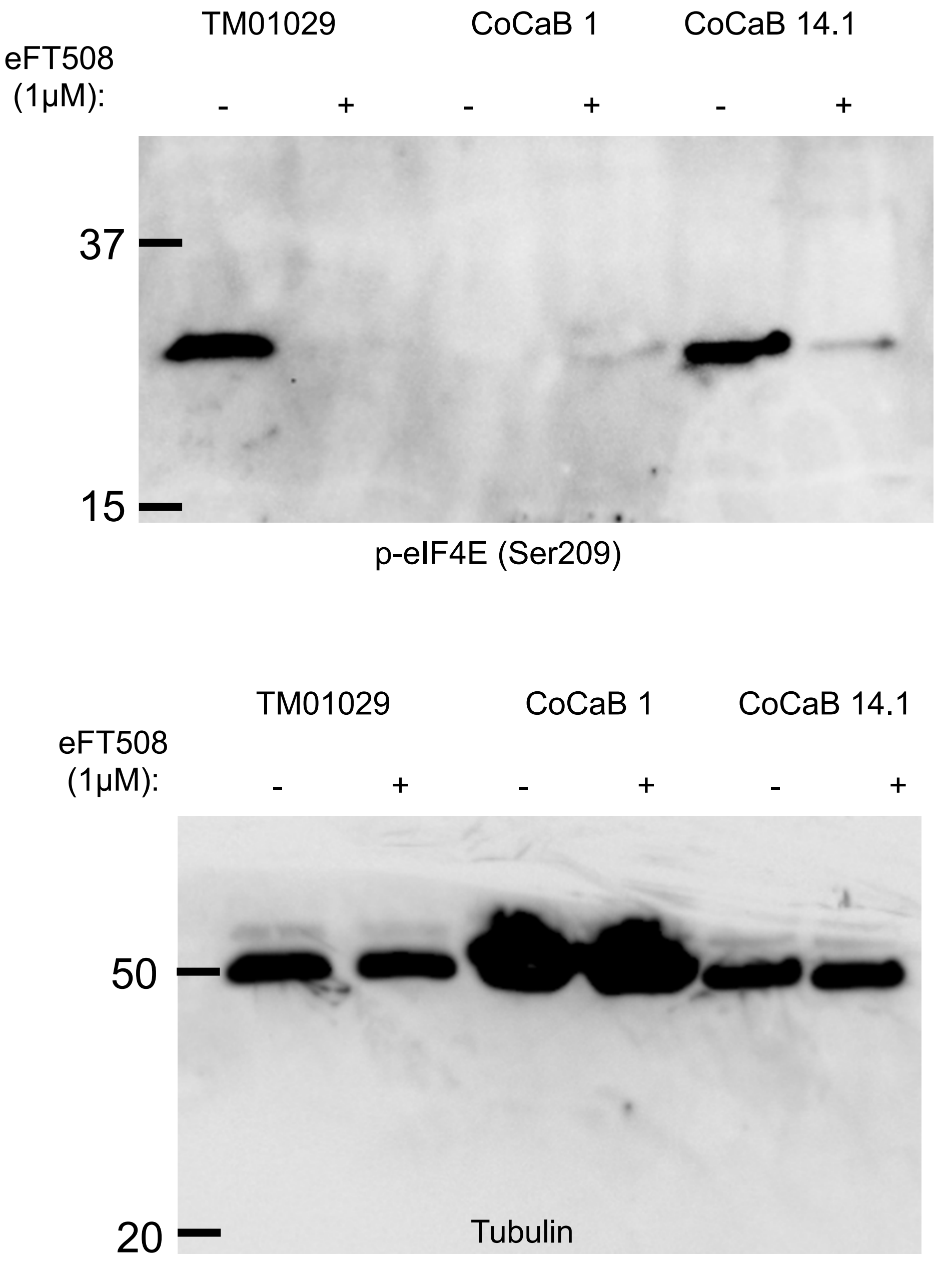
TM01029
CoCaB 1
CoCaB 14.1
eFT508
(1µM):
- + - + - +
37
15
p-eIF4E (Ser209)
TM01029
CoCaB 1
CoCaB 14.1
eFT508
(1µM):
- + - + - +
50
20
Tubulin

## Supplemental Figure 3C

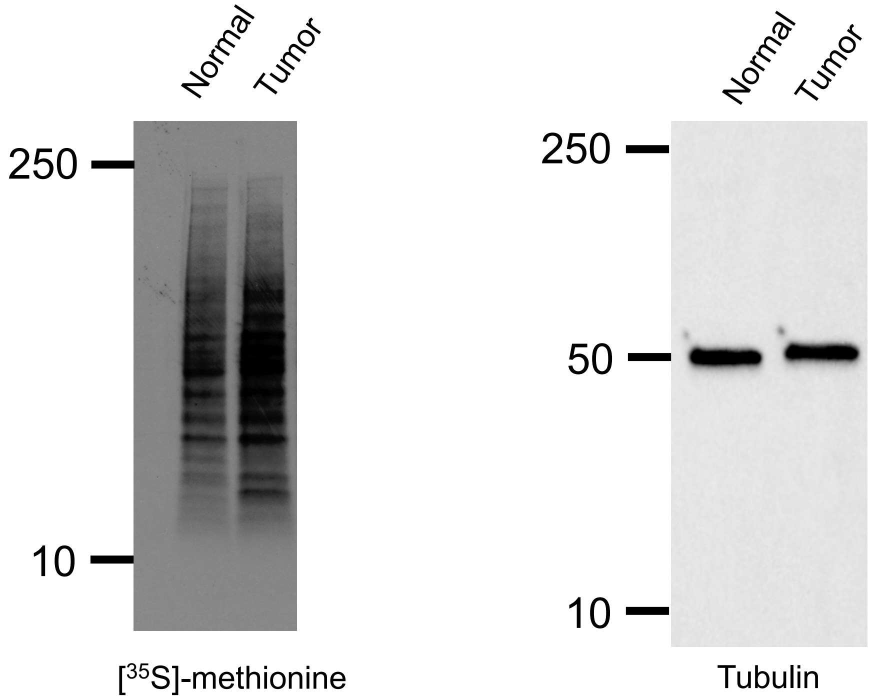

## Supplemental Figure 5

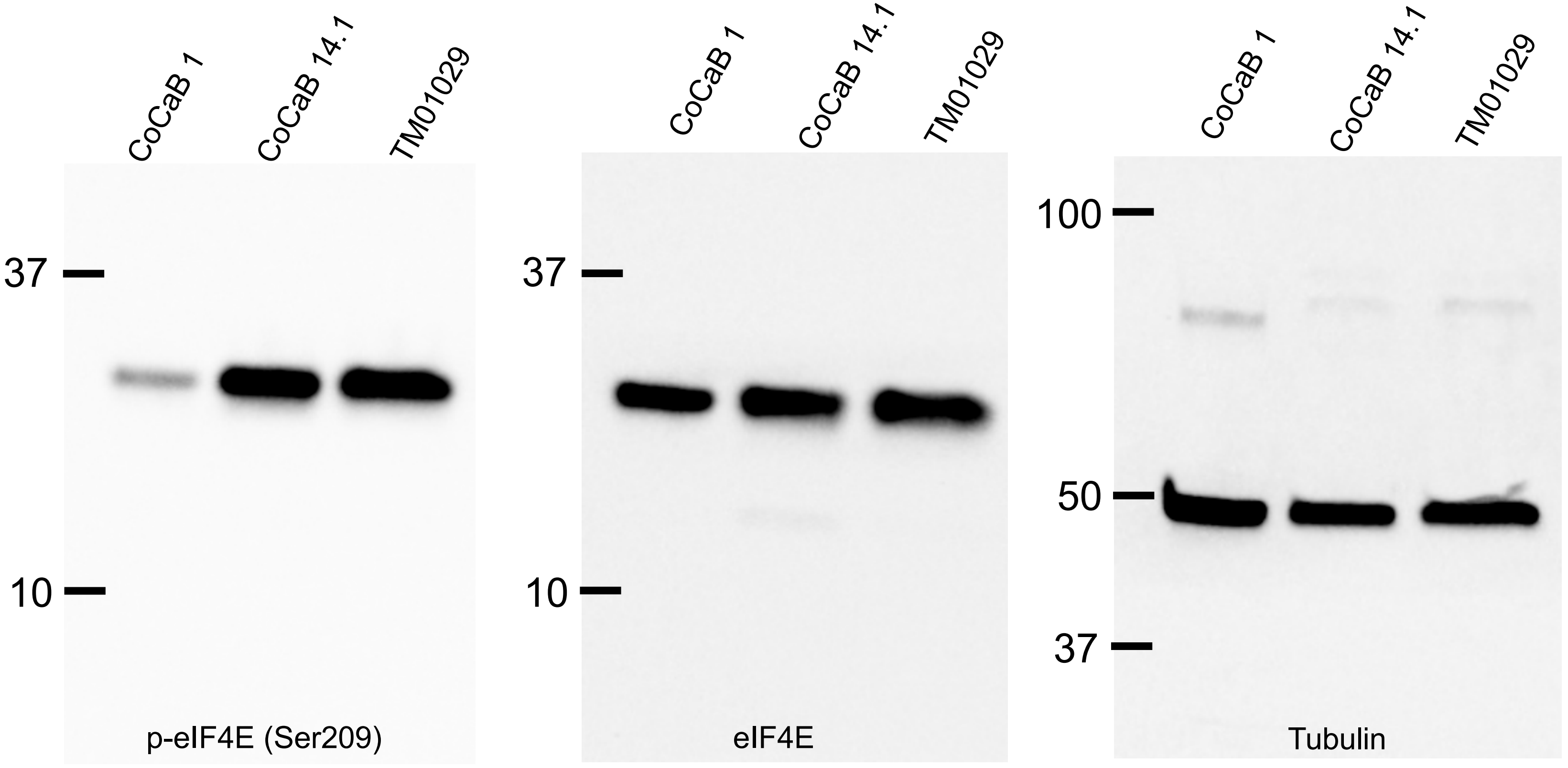

Supplement: Supplemental data [file jciinsight-6-144920-s014.pdf]
